# Indications for breast magnetic resonance imaging at a referral center for the diagnosis and treatment of breast cancer in Brazil

**DOI:** 10.1590/0100-3984.2019.0114

**Published:** 2021

**Authors:** Samuel Silva Ferreira, Adriene Moraes Campos, Patrícia Lima Fernandes, Izabela Machado Pereira, Flavia Maria Rodrigues, Antônio Fernando Braga Ferreira Victor, Ivie Braga de Paula

**Affiliations:** 1 Hospital Felício Rocho - Radiologia, Belo Horizonte, MG, Brazil.; 2 FAME - Faculdade de Medicina de Barbacena, Barbacena, MG, Brazil.

**Keywords:** Breast neoplasms/diagnostic imaging, Magnetic resonance imaging, Early detection of cancer/methods, Neoplasias de mama/diagnóstico por imagem, Ressonância magnética, Detecção precoce de câncer/métodos

## Abstract

**Objective:**

To describe the indications for breast magnetic resonance imaging (MRI) at a referral center for breast cancer in Brazil.

**Materials and Methods:**

This was a retrospective study in which we reviewed the clinical records, including physician requests and patient questionnaires, of women who underwent breast MRI between 2014 and 2018 at a referral center for the diagnosis and treatment of breast cancer in the city of Belo Horizonte, Brazil.

**Results:**

The most common indication for breast MRI was as a complement to mammography/ultrasonography (in 43.6% of cases), followed by breast cancer staging (in 25.1%), the screening of patients at high risk (in 17.4%), the evaluation of breast implants (in 10.1%), and the evaluation of the response to neoadjuvant chemotherapy (in 3.8%).

**Conclusion:**

Although there is strong evidence supporting the use of breast MRI, mainly for the screening of high-risk patients, the imaging method is underutilized in Brazil.

## Abstract (pt)

**Objetivo:**

Este estudo tem como objetivo descrever o perfil de indicações de ressonância magnética (RM) das mamas em um hospital referência em câncer de mama.

**Materiais e Métodos:**

Estudo descritivo retrospectivo de solicitações médicas de exames e questionários de mulheres que realizaram RM das mamas em um centro de referência no diagnóstico e tratamento do câncer de mama no período de 2014 a 2018.

**Resultados:**

A indicação mais frequente foi complemento de mamografia/ultrassonografia (43,6%), seguida por estadiamento do câncer de mama (25,1%), rastreamento de pacientes de risco elevado (17,4%), avaliação de implantes (10,1%) e avaliação de resposta a tratamento por quimioterapia neoadjuvante (3,8%).

**Conclusão:**

Embora existam fortes evidências em favor do uso da RM das mamas, principalmente para rastreamento em pacientes de alto risco, o método ainda é subutilizado no nosso meio.

## INTRODUCTION

Breast cancer is one of the most common neoplasms in women, second only to nonmelanoma skin cancer. In Brazil, it is estimated that there will be 59,700 new cases each year in 2018 and 2019^(1)^.

Mammography plays a well-established role in the screening and diagnosis of breast cancer, being capable of detecting the disease in its initial stages and, in conjunction with the proper treatment, reducing the mortality rate by approximately 60%^(2)^.

Ultrasound is not included as a screening method for breast cancer in most international guidelines, being considered a complementary imaging method, especially in women at high risk and in those with dense breasts, as well as a diagnostic evaluation tool in patients under 40 years of age.

Magnetic resonance imaging (MRI) has been utilized to detect breast cancer for more than 25 years. It presents higher sensitivity than does mammography, with a detection rate of over 90%, although its specificity is variable, ranging from 30% to 90%^(3)^. In the evaluation of the *in situ* component, MRI presents higher sensitivity than do mammography and ultrasound, with advantages in the diagnosis of lesions in which the nuclear grade of the cancer cells is high; that is, in the detection of biologically relevant cancer^(4,5)^. The disadvantages of MRI are its high cost in comparison with other methods and its limited availability in Brazil.

MRI can be utilized for breast cancer screening or as a complement to mammography or ultrasound for diagnostic clarification. Although its role in screening high-risk patients is well established, that indication, albeit increasingly more common, still does not account for the majority of examinations performed in the United States^(6)^. There are few data regarding the indications for breast MRI in Brazil, which indicates how slowly this imaging method is being incorporated into the treatment of patients in the country.

The objective of the present study was to describe the indications for breast MRI at a referral center for breast cancer in Brazil.

## MATERIALS AND METHODS

This was a descriptive retrospective study, in which we reviewed clinical records, including physician requests for breast MRI, at a referral center for the diagnosis and treatment of breast cancer between 2014 and 2018. We used Google Docs to collect data from the records. The data collected were then imported into and analyzed with the SPSS Statistics software package, version 23 (IBM Corp., Armonk, NY, USA).

## RESULTS

We evaluated 1,353 breast MRI examinations. The most common indication, registered in 590 (43.6%) of the examinations, was as a complement to mammography/ultrasound. Additional indications for breast MRI were breast cancer staging, in 339 (25.1%) of the cases; the screening of high-risk patients for breast cancer, in 236 (17.4%); the evaluation of breast implants, in 136 (10.1%); and the evaluation of the response to neoadjuvant chemotherapy, in 52 (3.8%).

If the indication for breast MRI was unclear, we searched for information in the questionnaires to understand the intent of the physician request and consequently included the indication in one of the subgroups previously described. It is noteworthy that all the patients who undergo breast MRI in the radiology department of our institution are personally interviewed immediately before the examinations by physicians, who evaluate their questionnaires.

## DISCUSSION

The indications for breast MRI examinations at our institution are similar to those found at referral centers in the United States, where it is used as a complement to other imaging methods in 40.3% of cases, for the screening of high-risk patients in 31.7%, and for the staging of breast cancer in 16.2%, other indications accounting for 11.8%^(6)^.

**Figure 1:**
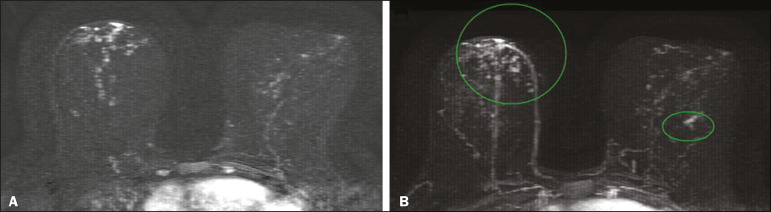
A 46-year-old patient with Cowden syndrome who presented with a BI-RADS category 2 finding (bilateral cysts) on ultrasound and a BI-RADS category 3 finding (punctiform calcification foci in the lower inner quadrant and central region of the right breast) on mammography. Breast MRI showed an area of non-nodular enhancement with segmental distribution in the central region of the right breast, extending to the upper inner quadrant, corresponding to the calcifications observed on mammography. A focal area of non-nodular enhancement can also be seen between the upper and lower outer quadrants of the left breast, with na isointense signal in the T2-weighted sequences and a hyperintense signal in the diffusion-weighted sequence with a high b-value. Biopsies were performed in both breasts, and the pathology findings were consistent with bilateral intermediate grade ductal carcinoma *in situ*.

There are ample data in the literature about the indications for breast MRI^(8-13)^, the level of evidence being highest for the screening of high-risk patients; that is, women who present a risk greater than 20% of developing breast cancer in their lifetime. That group includes women with BRCA gene mutations, those who underwent thoracic radiotherapy for lymphoma at 10-30 years of age, those with syndromes associated with breast cancer, such as the Li-Fraumeni and Cowden syndromes (Figure 1), and those with a strong family history that puts them in the risk group according to the calculation performed in existing risk models such as the Gail and Tyrer-Cuzick models.

**Figure 2:**
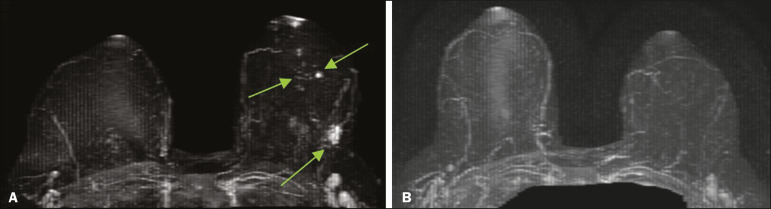
A 49-year-old patient diagnosed with invasive ductal carcinoma in the left breast. A: MRI showing multifocal lesions, with irregular nodules and foci of enhancement in the left breast, consistent with a diagnosis of neoplasm. B: MRI performed six months later, after the end of neoadjuvant chemotherapy, indicating that of the lesions in left breast had disappeared, consistent with an imaging complete response to the chemotherapy.

In patients already diagnosed with breast cancer, MRI detects additional ipsilateral sites in 6-34% of cases and in the contralateral breast in 4-24%^(12)^. The use of MRI in the preoperative staging of breast carcinoma, albeit controversial, has been shown to be beneficial in cases of aggressive carcinomas in dense breasts, in which mammography is less sensitive, in the evaluation of lobular carcinoma due to increased risk of multifocal and multicentric distribution, and in cases of extensive ductal carcinoma *in situ*. In the evaluation of patient response to neoadjuvant chemotherapy (Figure 2), MRI correlates well with the pathology findings in the surgical specimen.

**Figure 3:**
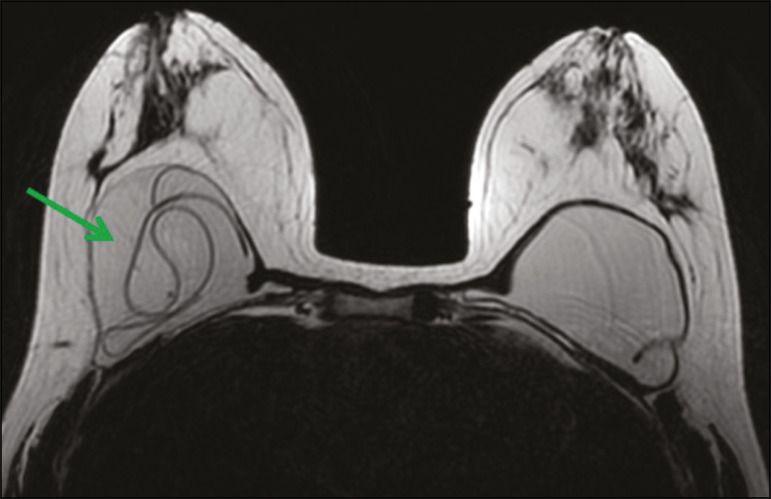
A 48-year-old patient submitted to breast MRI to evaluate the integrity of breast implants. Axial inversion recovery MRI sequence with water suppression, showing multiple hypointense irregular curvilinear lines in the right breast, consistent with the "linguine sign", which indicates intracapsular rupture.

In the investigation of the rupture of a prosthesis or implant, as well as of other complications related to breast augmentation (Figure 3), MRI is more effective than are other imaging methods, with a sensitivity and specificity above 90% for ruptures^(13)^.

For the detection of occult breast carcinoma in patients with metastatic disease in axillary lymph nodes and no indication of primary breast lesions on images obtained by other methods, MRI can be useful. Several studies have shown that MRI can detect primary breast tumors in most women who present with adenocarcinoma that has metastasized to the axillary lymph nodes^(13)^.

**Figure 4:**
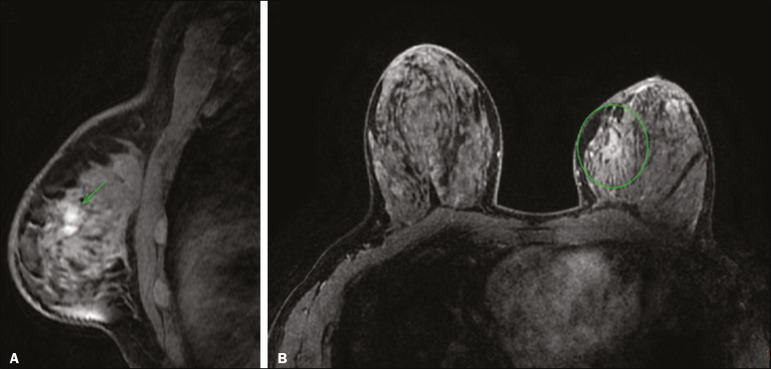
A 48-year-old patient recently diagnosed with invasive lobular carcinoma and lobular carcinoma in situ in the left breast, showing positive margins in the surgical specimen from breast-conserving surgery. MRI showing areas of non-nodular enhancement along the surgical margins in the left breast.

Most MRI examinations are performed as a complement to mammography or ultrasound. Although its use is controversial in various situations, including that in which it is used as a substitute for a more thorough evaluation with an imaging modality of lower technical quality, MRI can corroborate the decision to monitor any suspicious lesions after the diagnostic assessment with mammography and ultrasound is complete. However, it should not be an alternative to the biopsy of suspicious lesions or of clustered calcifications^(14,15)^. Other indications for breast MRI include special situations, such as the evaluation of pathological nipple discharge, the extent of a lesion in Paget's disease, the study of a residual lesion after breast-conserving surgery with positive margins, and the need for an auxiliary method to differentiate between postoperative alterations and tumor recurrence (Figure 4).

The profile of the patients described in this article could represent a selection bias, because the examinations were performed at the imaging clinic of a referral center for the diagnosis and treatment of breast cancer, where preoperative examinations are more common than they are at outpatient imaging clinics. In addition, most of the requesting physicians who referred patients to our clinic were themselves specialists in the treatment of breast cancer.

## CONCLUSION

Although there is considerable evidence supporting the use of breast MRI, the method is still underutilized in Brazil, not only for screening women considered to be at high risk but also for staging the disease and for evaluating the response to neoadjuvant chemotherapy. The profile of indications shows that the screening of high-risk patients still accounts for only a small portion of the examinations performed, despite the fact that the level of evidence is highest for that indication. Most breast MRI examinations are performed in order to clarify mammography or ultrasound findings, exactly the situation in which the application of MRI is most controversial.

New data on the use of MRI in the routine screening of high-risk patients are coming to light as the method becomes more accessible. Evidence on its use in pretreatment planning, in which it can improve staging, and in the characterization of tumor profiles (as more or less favorable) point to future directions for its application.
